# Characterization of a novel CSF1R mutation causing hereditary diffuse leukoencephalopathy with spheroids in a case presenting with young-onset dementia

**DOI:** 10.1192/bjo.2021.345

**Published:** 2021-06-18

**Authors:** Walid Nasr, Mahmoud Gad, Tareq Qassem

**Affiliations:** 1 Al-Amal Hospital, Ministry of Health and Prevention; 2 Maudsley Health, Al-Amal Hospital, Ministry of Health and Prevention, Mohammed Bin Rashid University Of Medicine and Health Sciences, Institute of Psychiatry, Ain Shams University

## Abstract

**Objective:**

This poster aims to report an unregistered mutation CSF1R gene in a patient presenting young-onset dementia.

Hypothesis: Novel heterozygous deletion–insertion mutation in the Colony-Stimulating Factor 1 Receptor (CSF1R) gene is linked to a case of hereditary diffuse leukoencephalopathy with spheroids (HDLS), presenting with young-onset dementia.

**Background:**

CSF1R mediates proliferation, differentiation, and survival of monocytes/ macrophages and microglia. Pathogenic variants in the CSF1R gene cause autosomal dominant diffuse hereditary leukoencephalopathy with spheroids characterized by variable behavioural, cognitive, and motor changes, usually presenting with young-onset dementia. The average lifespan after the start of the symptoms is often 6 years.

**Case report:**

Molecular genetic analysis of whole-exome sequencing (WES) was carried out for a 49-year-old male patient presenting with rapid cognitive decline, behavioural symptoms and impaired sphincteric control.

**Discussion:**

WES identified the heterozygous deletion–insertion variant c.2356_2357delinsAC p.(Leu786Thr) (chr5:149435867-49435868; hg19) in the CSF1R gene. To the best of our knowledge the variant has not been described in the literature so far (HGMD 2019.3). No allele frequencies in the general population have been documented.

**Conclusion:**

We believe that we have identified a novel mutation in the CSF1R gene. This mutation is likely to be linked to this patient presenting with young-onset dementia.

